# Subcutaneous Administration of Low-Molecular-Weight Heparin to Horses Inhibits Ex Vivo Equine Herpesvirus Type 1-Induced Platelet Activation

**DOI:** 10.3389/fvets.2018.00106

**Published:** 2018-05-28

**Authors:** Tracy Stokol, Priscila B. S. Serpa, Marjory B. Brooks, Thomas Divers, Sally Ness

**Affiliations:** ^1^Department of Population Medicine and Diagnostic Sciences, College of Veterinary Medicine, Cornell University, Ithaca, NY, United States; ^2^Department of Clinical Sciences, College of Veterinary Medicine, Cornell University, Ithaca, NY, United States

**Keywords:** EHV-1, P selectin, thrombin generation, unfractionated heparin, heparin

## Abstract

Equine herpesvirus type 1 (EHV-1) is a major cause of infectious respiratory disease, abortion and neurologic disease. Thrombosis in placental and spinal vessels and subsequent ischemic injury in EHV-1-infected horses manifests clinically as abortion and myeloencephalopathy. We have previously shown that addition of heparin anticoagulants to equine platelet-rich plasma (PRP) can abolish *ex vivo* EHV-1-induced platelet activation. The goal of this study was to test whether platelets isolated from horses treated with unfractionated heparin (UFH) or low-molecular-weight heparin (LMWH) were resistant to *ex vivo* EHV-1-induced activation. In a masked, block-randomized placebo-controlled cross-over trial, 9 healthy adult horses received 4 subcutaneous injections at q. 12 h intervals of one of the following treatments: UFH (100 U/kg loading dose, 3 maintenance doses of 80 U/kg), 2 doses of LMWH (enoxaparin) 80 U/kg 24 h apart with saline at the intervening 12 h intervals, or 4 doses of saline. Blood samples were collected before treatment and after 36 h, 40 h (4 h after the last injection) and 60 h (24 h after the last injection). Two strains of EHV-1, Ab4 and RacL11, were added to PRP *ex vivo* and platelet membrane expression of P selectin was measured as a marker of platelet activation. Drug concentrations were monitored in a Factor Xa inhibition (anti-Xa) bioassay. We found that LMWH, but not UFH, inhibited platelet activation induced by low concentrations (1 × 10^6^ plaque forming units/mL) of both EHV-1 strains at 40 h. At this time point, all horses had anti-Xa activities above 0.1 U/ml (range 0.15–0.48 U/ml) with LMWH, but not UFH. By 60 h, a platelet inhibitory effect was no longer detected and anti-Xa activity had decreased (range 0.03 to 0.07 U/ml) in LMWH-treated horses. Neither heparin inhibited platelet activation induced by high concentrations (5 × 10^6^ plaque forming units/mL) of the RacL11 strain. We found substantial between horse variability in EHV-1-induced platelet activation at baseline and after treatment. Minor injection site reactions developed in horses given either heparin. These results suggest that LMWH therapy may prevent thrombotic sequelae of EHV-1, however further evaluation of dosage regimens is required.

## Introduction

Equine herpes virus type 1 (EHV-1) is a ubiquitous equine pathogen that causes outbreaks of respiratory disease, abortion and neurologic disease worldwide (1–7). The virus is acquired through inhalation of infected respiratory secretions and, after replicating in the respiratory mucosa and regional lymph nodes, establishes a cell-associated viremia (8, 9). Infection of the endothelium is facilitated by the adhesion of viral-infected leukocytes (10, 11) and leads to a vasculitis with subsequent tissue infection and injury. The resulting tissue damage, from a combination of inflammation and ischemia, leads to the syndromes of abortion and myeloencephalopathy (7, 12, 13).

On histological examination, thrombi can be found in the blood vessels of EHV-1-infected horses (12–14). This finding, with the evidence of ischemic injury in the placenta and spinal cord, indicates that activation of hemostasis is involved in the pathogenesis of abortion and neurologic disease. In one study of horses experimentally infected with EHV-1, some horses had high plasma D-dimer concentrations during the viremic phase of infection (15), providing indirect evidence of a hypercoagulable state. The cause of this hypercoagulable state is unknown but is likely multifactorial. Direct endothelial injury exposing subendothelial matrix proteins and tissue factor could activate platelets and coagulation, respectively. Tissue damage from vasculitis or inflammatory cytokines from viremia or tissue inflammation can upregulate procoagulants and inhibit anticoagulant responses. We have also found that the virus itself can contribute to a hypercoagulable state by upregulating tissue factor in equine monocytes exposed to EHV-1 *ex vivo* (16). The virus can also directly activate coagulation and generate thrombin in equine plasma, presumably through tissue factor expression in the viral envelope (17). Both tissue factor and procoagulant phospholipids are thought to be acquired from the host cell membranes during replication (18). Virus-mediated thrombin generation in equine plasma can then activate platelets (17), which could contribute to thrombus formation.

Heparin-based anticoagulants, including unfractionated heparin (UFH) and low-molecular-weight heparin (LMWH), have been used for thromboprophylaxis in horses with various disorders, including colic and laminitis post-surgical colic (19, 20). The pharmacokinetic profiles for various doses of UFH and LMWH have been described, with anticoagulant activities generally peaking between 4–6 h after administration of a single dose (21–24). Compared to UFH, LMWH is considered to have a more predictable pharmocokinetic profile and is associated with fewer side-effects, including swelling at injection sites and red blood cell agglutination (19, 25–29).

We have recently reported that UFH and LMWH can inhibit EHV-1-induced platelet activation when spiked in equine platelet-rich plasma (PRP) *ex vivo* (30). Activated platelets were quantified by the flow cytometric-based detection of surface P selectin, as a marker of α-granule release. The observed inhibition of EHV1-induced platelet activation *ex vivo* raises the possibility that heparin anticoagulants could be given to horses to reduce the incidence of abortion and myeloencephalopathy in EHV-1 outbreaks. This is important because there are currently no treatments that can be given to horses minimize these clinical syndromes. Indeed, a recent experimental trial showed that early administration of the antiviral drug, valicyclovir, did not change the incidence of neurological symptoms, although the severity of symptoms was reduced (31). Thus, the goal of this study was to extend our experiments using *ex vivo* spiked equine PRP to determine whether UFH and LMWH administered at commonly used clinical doses to horses could inhibit EHV-1-induced platelet responses in PRP *ex vivo*.

## Materials and Methods

### Drug Treatment and Blood Sample Collection

**Figure 1:**
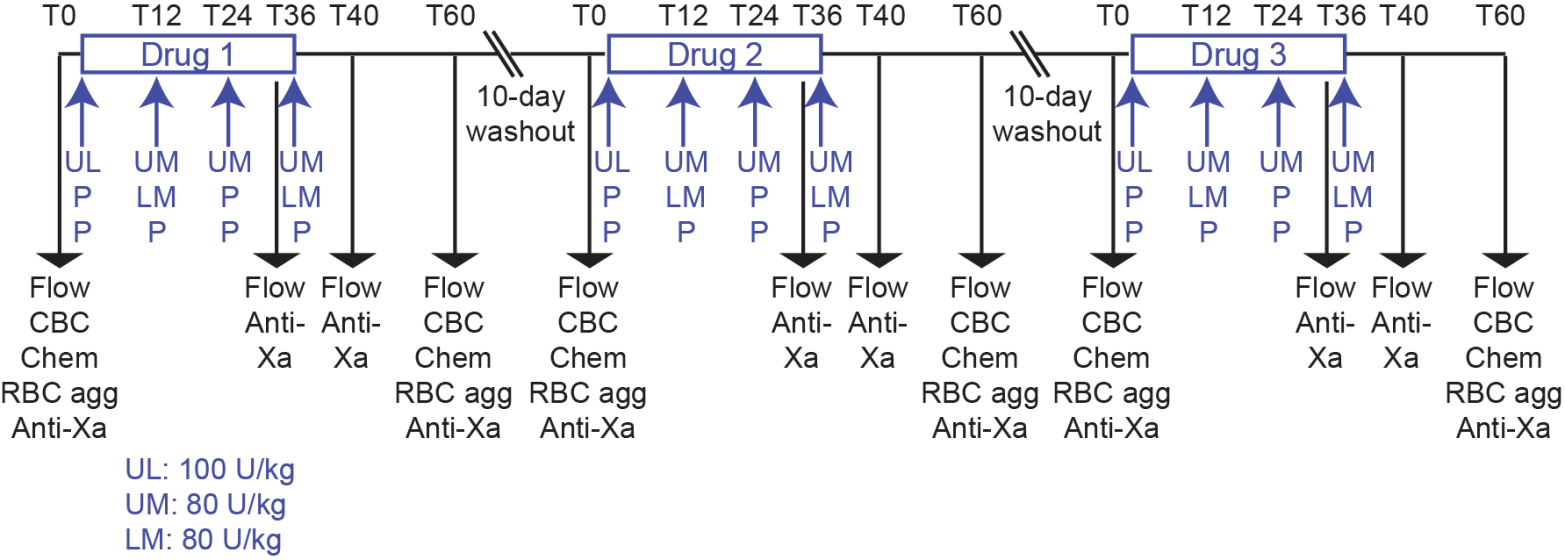
Experimental design for a randomized heparin placebo-controlled anticoagulant drug trial in 9 horses. The order of drug administration in individual horses was block-randomized by personnel not involved in drug administration or data acquisition or analysis. Drugs or placebo were administered for 2 days at 12 h intervals with blood samples being taken for testing before treatment (T0) and 36, 40 and 60 h after the start of treatment. A 10 day washout period separated drug treatments. The 36, 40 and 60 h time points corresponded to times just before, 4 h after and 24 h after the last subcutaneous injection of the drug or placebo. For UFH, the drug was given as a loading dose of 100 U/kg, then 3 maintenance doses of 80 U/kg were given at 12 h intervals (4 total doses). For LMWH, the drug was given at a dose of 80 U/kg every 24 h (2 doses), with placebo administration at the intervening 12 h intervals (2 doses). The timing of the initiation of UFH and LMWH treatment was staggered to ensure that samples for blood collection and subsequent measurements were consistent in relation to the last dose. Placebo was given every 12 h (4 doses). Blood samples were collected at 0 and 60 h for flow cytometric evaluation of platelet activation (measurement of P selectin expression after exposure to agonists *ex vivo*; Flow), measurement of drug inhibitory activity with an anti-Xa bioassay (anti-Xa), automated hemogram analysis (CBC), evaluation for red blood cell agglutination (RBC agg) and measurement of select biochemical analytes (Chem). Additional blood samples were collected at 36 and 40 h for flow cytometric evaluation of platelet activation and anti-Xa inhibitory activity.

In this block-randomized double-blinded placebo-controlled cross-over study, 9 horses were administered UFH (Heparin sodium injection, multi-dose vials, Sagent Pharmaceuticals Inc, Schaumburg, IL, USA), LMWH (Enoxaparin sodium injection, multi-dose vials, Sanofi-Aventis US LLC, Bridgewater, NJ, USA) or placebo (0.9% sodium chloride injection USP, Baxter Healthcare Corporation, Deerfield, IL, USA) subcutaneously, followed by a 10 day washout between treatments (Figure 1). The number of horses was based on a power analysis with a free on-line tool (power and sample size.com), using a 2-sided equality estimate assuming a partial or 50% inhibitory response, with a SD of 25%, power of 0.80 and type 1 error at 5%. The horses were group housed at the Equine Park at Cornell University and were clinically healthy. They consisted of 2 geldings and 7 mares, 16–24 years of age. Breed distribution included 2 Quarter Horses, 2 Thoroughbreds, 2 Warmbloods, 2 Oldenburgs and 1 Holsteiner. The horses ranged in weight from 511 to 626 kg. The horses were divided into 2 groups consisting of 4 and 5 horses to facilitate treatment and data acquisition. Personnel, who were not involved in administering the drugs, data acquisition, or data analysis, randomized the order of treatment. Investigators were blinded as to each treatment until the conclusion of the study. Unfractionated heparin was given at a loading dose of 100 U/kg followed by 3 maintenance doses of 80 U/kg every 12 h (4 total doses). Low-molecular-weight heparin was administered at a dose of 80 U/kg every 24 h (2 doses), with placebo being given at the intervening 12 h intervals to maintain blinding. Placebo was injected every 12 h for 4 total doses. Sequential injections were administered on alternate sides of the neck at different sites (upper or lower). The chosen doses were based on currently used treatment protocols at our institution for UFH, which are in the midrange of prior reports (19, 21, 29), and the high dose used in the original pharmacokinetic studies of LWMH (23). Because UFH and LMWH were administered twice and once daily, respectively, the first dose of each drug was given at different times to ensure consistency in the timing of sample collection and data acquisition in relation to the last administered dose (Figure 1).

For flow cytometric testing of platelet activation and measurement of anticoagulant activity, blood was collected from the jugular vein via an 18 G needle (Monoject veterinary hypodermic needle, Covidien Ltd, Mansfield, MA, USA) into 6 ml syringes (Monoject syringe, Covidien Ltd) prefilled with 3.8% citrate anticoagulant (Sigma-Aldrich, St Louis, MO, USA), maintaining a ratio of 9 parts blood to 1 part citrate. The following time points were collected: Before treatment (baseline, 0 h), before the final dose (36 h after the start of treatment), and 4 and 24 h after the final dose (40 and 60 h after the start of treatment) (Figure 1). The 40 h time point was chosen to obtain a sample at or close to peak anticoagulant activities. The 36 and 60 h time points were chosen to obtain samples where drug concentrations were expected to be at trough anticoagulant levels after a single dose (21–24). To minimize platelet activation during collection, the needle was inserted first and blood allowed to drip for a few seconds before attachment of the syringe and sample collection. The required volume was then drawn slowly and smoothly into the syringe and the samples were transported carefully, minimizing agitation, to the laboratory. To monitor horses for anemia, a known effect of heparin therapy (19, 25–28) or other complications, blood samples were collected from the jugular vein into EDTA- and non-anticoagulant vacutainers (Becton-Dickinson, Franklin Lakes, NJ, USA) with a vacutainer needle (21 G, Becton-Dickinson Eclipse™ Vacutainer® with pre-attached holder). The EDTA blood and serum were used for clinical pathologic testing and assessment for red blood cell agglutination at 0 and 60 h. Horses were also examined for reaction site swelling and clinically obvious hemorrhage before each treatment or blood sampling point and any reactions were recorded.

### Ex Vivo Platelet Activation and Flow Cytometric Detection of P Selectin Expression

All reagents were from Sigma-Aldrich, unless otherwise specified. We used flow cytometric expression of P selectin as our marker of platelet activation (17, 30). Within 45–60 min of collection, citrate-anticoagulated blood was gently expelled into a 15 ml polypropylene tube and red blood cells were allowed to settle by gravity sedimentation for 20 min at room temperature (20–23°C). The leukocyte-platelet-rich plasma was removed and centrifuged at 250 *× g* at 21°C for 10 min to obtain platelet-rich plasma (PRP). The PRP was then allowed to “rest” at room temperature for 30 min. Then, the PRP was diluted in platelet- buffer (10 mM HEPES and 140 mM sodium chloride, pH 7.4), supplemented with 1 mM gly-pro-arg-pro-NH_2_ (GPRP) and 2.5 mM calcium chloride, and exposed to the following agonists for 10 min at 37°C: Thrombin at low and high concentrations of 0.15 and 0.5 U/ml, respectively, as a heparin-sensitive positive control, adenosine 5′-diphosphate (ADP, 40 µM, Bio/Data Corporation, Horsham, PA, USA) as a heparin-resistant positive control, phosphate-buffered saline (PBS) as a negative virus control and two strains of EHV-1, Ab4 and RacL11, at low and high concentrations equivalent to 1 × 10^6^ plaque forming units (PFU)/mL and 5 × 10^6^ PFU/ml, respectively. The Ab4 and RacL11 strains were propagated in rabbit kidney 13 and equine kidney cells, respectively, and isolated from cell lysates on a sucrose cushion via ultracentrifugation as we have previously described (17). One harvested isolate of each strain was used for the entire study.

In our previous studies, we had standardized the amount of EHV-1 that was added to the PRP to a defined platelet concentration, i.e., PFU/platelet (17, 30). When this protocol was used for the first group of horses given the first dose of drug, we saw no inhibition of thrombin- or EHV-1-induced platelet activation, particularly 4 h after the last dose of drug, when the drugs would be expected to be exerting the maximal inhibitory effect after subcutaneous administration (21–24). By chance alone, we expected at least 1 of the 4 horses to have been given one of the heparin anticoagulants and the lack of any inhibitory effect prompted us to re-evaluate our experimental flow cytometric protocol for measuring *ex vivo* platelet activation. When we examined our previously used protocol, we realized that in order to standardize the EHV-1 concentration to a set platelet number, we were diluting the PRP between 1:30 and 1:150, depending on the platelet count in the PRP. Since EHV-1 appears to activate platelets through tissue factor-triggered thrombin generation (17), we reasoned that this dilution would abolish any inhibitory effect of the administered drug on platelet activation. In addition, the EHV-1-induced activation response is dependent on coagulation factors (and inhibitors) in PRP and not on the platelet count *per se*. Thus, we modified our protocol to expose a standard volume of PRP (20 µl in a 100 µl reaction volume representing a 1:5 dilution with platelet buffer) to a standard concentration of virus, which was equivalent to PFU/mL versus PFU/cell. The chosen PRP dilution was based on the dilution used for testing of the anticoagulant activity of heparin with an activated Factor X inhibition bioassay (anti-Xa activity). This higher amount of PRP necessitated the inclusion of more GPRP than that used previously [1 mM versus 0.2 mM (17)] to inhibit fibrin polymerization in the reaction solution. Preliminary testing of the PRP from 2 horses not enrolled in the study showed that platelets were variably activated by thrombin, ADP and EHV-1 at the chosen concentrations and we proceeded with this revised protocol with the second group of 5 horses, repeating treatment 1 (still blinded as to the treatment and after a 10 day washout) in the first group of 4 horses.

After incubating PRP for 10 min with the various agonists, labeling to detect P selectin expression was performed by the addition of an Alexa647-conjugated antibody against P selectin (final concentration of 66 ng/ml of clone Psel.KO.2.7, Novus Biologicals LLC, Littleton, CO, USA). Samples were labeled for 10 min at room temperature in the dark. The reaction was quenched with 0.6 ml of platelet buffer containing 0.2 mM supplemental GPRP and 2.5 mM calcium chloride and the samples were immediately analyzed with a flow cytometer (BD FACSCalibur, BD Biosciences, San Jose, CA, USA). For analysis, platelets were gated on their forward and side scatter characteristics and the percentage of P selectin-positive platelets determined from histogram plots, as we have previously described (30).

### Measurement of the Anticoagulant Activity of Unfractionated and Low-Molecular-Weight Heparin

The anticoagulant activity (anti-Xa activity) of UFH and LMWH was measured in platelet-poor plasma (PPP) at the Comparative Coagulation Laboratory at Cornell University. The PPP was harvested from the supernatant of PRP after high-speed centrifugation (16,000 *× g*, Accuspin Micro, ThermoScientific, Rockford, IL, USA) for 5 min. The PPP was frozen at −20°C in a dedicated freezer and assays were performed in batch after each treatment for each group of horses with the other investigators remaining blinded as to the results. The PPP was thawed at 37°C in a water bath before analysis. The assay is configured with a bovine activated Factor X reagent added in excess to the test plasma and a chromogenic substrate of Factor Xa (Liquid anti-Xa, Diagnostica Stago, Parsipanny, NJ, USA). The assay is performed using the manufacturer’s automated coagulation analyzer (STA Compact, Diagnostica Stago). In this assay, residual uninhibited Factor Xa cleaves the chromogenic substrate so the inverse of the color change in the reaction mixture is proportional to the drug concentration in the test plasma. Results are expressed as U/mL anti-Xa, based on assay calibration with a standard containing known UFH or LMWH concentrations (STA-calibrators HBPM or LMWH, Diagnostica Stago).

### Thrombin Generation Assay

Thrombin generation assays were performed on baseline samples from all treatments on frozen PPP from a subset of 5 horses. The frozen samples were thawed at room temperature before analysis. For the assay, a 1:5 dilution of PPP was exposed to both virus strains at concentrations equivalent to the platelet activation assays. The virus served as the trigger for thrombin generation (no additional tissue factor or phospholipid was added) and PBS was used as a negative control. The cleavage of a fluorogenic thrombin substrate (containing calcium) was monitored in a spectrophotometer (Spectromax M3, Molecular Devices with Softmax Pro 6.2.1 software). The virus and diluted PPP (40 µL) were added in duplicate to wells of a black 96-well plate (Grenier, Bio-One GmbH, Fridenhausen, Germany), followed by the addition of the fluorogenic substrate/calcium reagent (60 µL, Technothrombin® TGA, Technoclone GmbH, Vienna, Austria) and the change in fluorescence, indicating the amount of thrombin generated, was measured kinetically every minute for 120 min using bichromatic wavelengths of 360/460 nm at 37°C. A human plasma-based control (Technothrombin® TGA C high, Technoclone GmbH) spiked with recombinant tissue factor (1:500 final concentration, Innovin, Dade-Behring) served as a positive assay control. All baseline PPP were run on the same plate in a single run from an individual horse and all samples were run in duplicate. The resulting raw fluorescent data was converted to nM of thrombin generated using proprietary software (Technothrombin® TGA Excel Software, Technoclone GmbH) and a predetermined calibration curve (Technothrombin® TGA Calibrator, Technoclone GmbN). The average results for the duplicates of time to peak thrombin (minutes), peak thrombin (nM), velocity index (rate of thrombin formation in nM/minute) and total thrombin generated or area under the curve (AUC, thrombin in nM x minute) were provided, along with thrombin generation curves. Due to a software error, the lag time was manually calculated from the converted data as the time taken from the start of analysis to when 4 nM or greater of thrombin was generated. For samples with little to no thrombin generation, the lag time was assigned a value of 60 min.

### Clinical Pathologic Testing

Automated hemogram results were obtained from EDTA-anticoagulated blood with a hematology analyzer (ADVIA 2120, Siemens Healthcare Diagnostics Inc., Tarrytown, NJ, USA). Modified Wright’s-stained blood smears were examined by trained medical technologists to detect platelet clumps and verify automated platelet counts. For assessment of red blood cell agglutination, the EDTA-anticoagulated blood was diluted 1:4 with 0.9% saline, then a drop of the diluted blood was placed on a glass slide and coverslipped and examined. Agglutination was recorded as being present or absent. Biochemical analysis for renal analytes (serum urea nitrogen and creatinine concentrations), liver enzymes (sorbitol dehydrogenase, glutamate dehydrogenase, aspartate aminotransferase and gamma glutamyl transferase activities), protein-related analytes (total protein, albumin, and globulin concentrations), and serum amyloid A concentrations was performed on serum with an automated chemistry analyzer (Hitachi P modular, Roche Diagnostics, Indianapolis, IN, USA) using manufacturer’s reagents, with the exception of sorbitol dehydrogenase (Sigma-Aldrich), glutamate dehydrogenase (Randox Laboratories Ltd, Antrim, UK) and serum amyloid A (LZ test Eiken-SAA, Mast House, Merseyside, UK).

## Statistical Analysis

Data had a non-Gaussian distribution and results are described and analyzed using non-parametric statistical methods and statistical software (Prism 6.0 for MacOS, version 6.0 f, GraphPad Software Inc, La Jolla, CA, USA). Medians of 2 groups were compared with a Wilcoxon-matched pairs signed rank test. Medians of more than 2 groups were compared using a Friedman’s test with Dunn’s correction for multiple comparison tests. Alpha was set at 0.05.

## 

## Results

**Figure 2:**
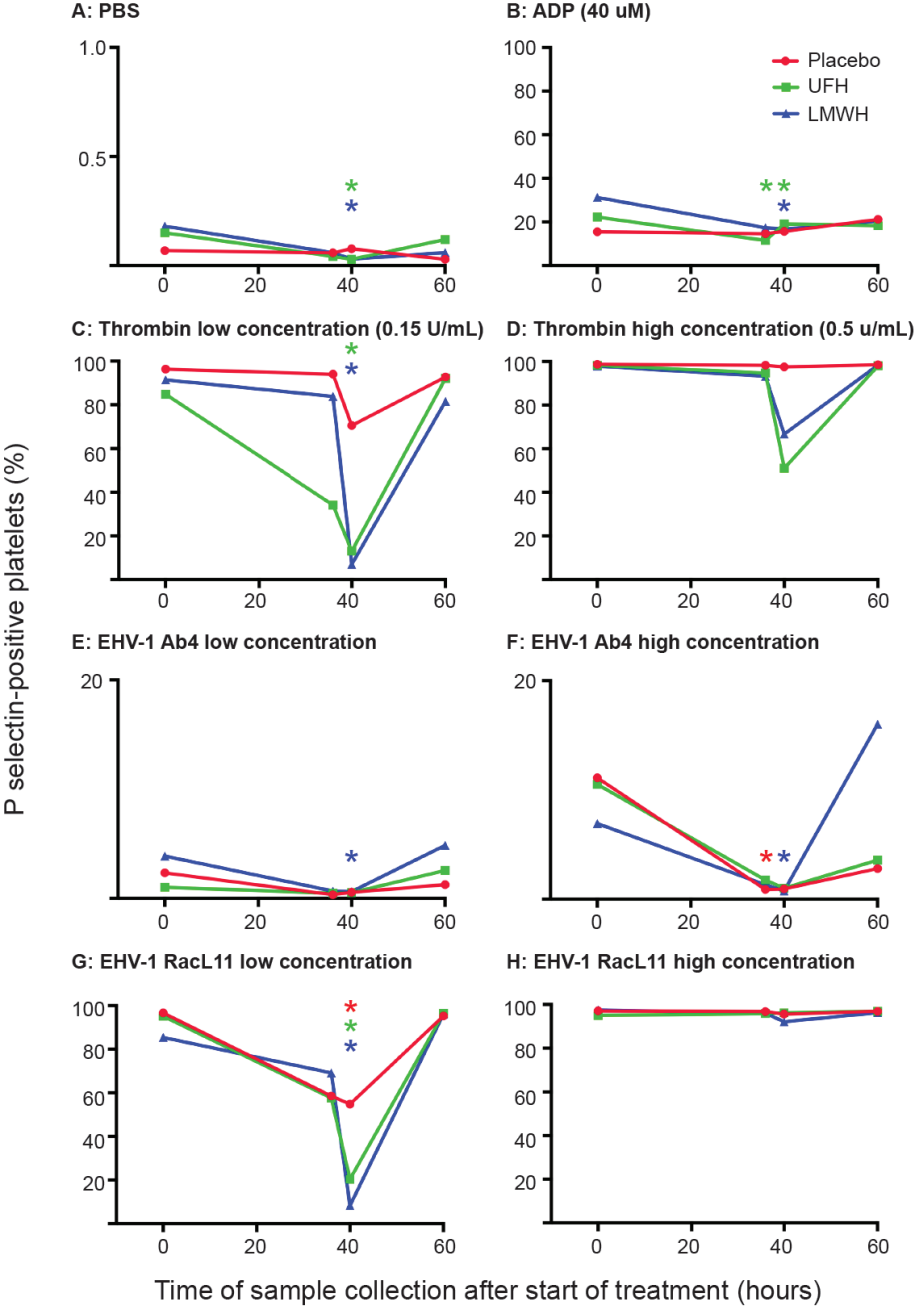
Changes in the median percentage of platelets expressing P selectin in platelet-rich plasma of 9 horses before treatment (0 h), 36 h (before the last dose), 40 h (4 h after the last dose) and 60 h (24 h after the last dose) of placebo (red), unfractionated heparin (UFH, green) or low-molecular-weight heparin (LMWH, blue). Platelets were exposed to PBS **(A)** as a negative control, ADP **(B)** as a positive control, low **(C)**, 0.15 U/ml) and high (**D**), 0.5 U/ml) concentrations of thrombin, or low (1 × 10^6^ PFU/ml) and high (5 × 10^6^ PFU/ml) concentrations of the EHV-1 strains, Ab4 **(E)**, low concentration; **(F)**, high concentration and RacL11 **(G)**, low concentration; **(H)**, high concentration for 10 min *ex vivo*. Note that the Y-axis scale bar is different for PBS and low and high concentrations of Ab4. *Colored stars represent significant changes for the relevant drug at each time point compared to 0 h (*p* < 0.05).

In LMWH-treated horses, a consistent decrease in *ex vivo* platelet activation in response to most viral and pharmacologic agonists was seen at 40 h (4 h after subcutaneous administration of the 2nd and last dose) (Figure 2). In contrast, a significant decrease in platelet activation was only seen in UFH-treated horses at this time point after *ex vivo* stimulation with the low concentration of the RacL11 strain and low concentrations of the thrombin and ADP agonists. The inhibitory effect of both heparins on agonist-induced platelet activation was no longer apparent at 60 h (24 h after the last drug dose) (Figure 2), indicating that the inhibitory effect of heparin on *ex vivo* platelet activation was short-lived.

Results of anti-Xa analyses revealed that the anticoagulant effect of LMWH was greater than UFH and that the highest anti-Xa activities for both drugs occurred at 40 h (Table 1). All horses treated with LMWH attained levels of anti-Xa activity associated with effective thromboprophylaxis (0.1–0.2 U/ml) or therapeutic anticoagulant action (0.3–0.7 U/ml) in human medicine (23) at 40 h. In contrast, only 2 of the UFH-treated horses attained anti-Xa activities in the prophylactic target range at this time. Higher than baseline anti-Xa activity was detected in the plasma of all LMWH-treated horses at 36 and 60 h (i.e., 24 h after the first and last dose) but these levels of anti-Xa activity were insufficient to inhibit agonist-induced platelet activation. Anti-Xa activity was inconsistently detected after UFH therapy, with only 4 and 1 of 9 the horses having detectable activity at 36 or 60 h, respectively.

**Table 1:** Median (and range) anti-Xa activity in citrate-anticoagulated platelet-poor plasma of 9 horses administered placebo, unfractionated heparin (UFH) and low-molecular-weight heparin (LMWH) over 2 days.

Treatment	Anti-Xa activity (U/mL)			
	Hours after starting treatment			
	0	36	40	60
Placebo	0 (0–0.1)	0 (0–0)	0 (0–0)	0 (0–0)
UFH	0.02 (0–0.04)	0.03 (0–0.06)	0.04* (0–0.13)	0.02 (0–0.04)
LMWH	0 (0–0.03)	0.03 (0.02–0.06)	0.4* (0.15–0.48)	0.04* (0.03–0.07)

The anti-Xa activity was measured before treatment (0 h), then at 36 h, 40 h (4 h after the last dose) and 60 h (24 h after the last dose) after starting treatment.

* p = 0.0001 for UFH, p < 0.0001 for LMWH versus 0 h.

After LMWH treatment, 6 of the 9 horses developed subcutaneous swellings after 1 or 2 injections. Two horses bled excessively at either 36 or 40 h (suspect jugular hematoma in one horse, which resolved within 24 h, and excessive venipuncture bleeding in another horse). After UFH treatment, injection site swellings developed in 6 of the 9 horses with one or more doses (3 horses had similar swellings after LMWH injection). None of the UFH-treated horses were observed to bleed excessively. With placebo treatment, subcutaneous swellings developed in 2 horses after one or two injections. One of these horses also appeared to bleed excessively from venipuncture at the 40 h time point. This was the same horse that had excessive venipuncture bleeding after LMWH injection. The subcutaneous swellings with all treatments resolved within 24–72 h and none were hot or painful.

One horse suffered from a superficial wound with an associated soft tissue swelling below the right hind fetlock just before the start of drug treatment 3 (LMWH). The swelling progressed to a cellulitis with resulting peripheral edema and fever. Trial drug administration was withheld and the horse was treated with anti-inflammatory (oral phenylbutazone) and antimicrobial (trimethoprim-sulfonamide) agents for 7 days. Three weeks later, after the inflammation had resolved, the horse was given treatment 3 and completed the study.

There were no significant changes in the median results of select hematologic tests of the 9 horses before and after each treatment (Table 2). One horse had a platelet count below established reference intervals at 4 of the 6 tested 0 or 60 time points for all drugs combined. Two other horses had a low platelet count (at 0 and 60 h for one horse and at 0 h only for the second horse) with the placebo treatment. Platelet clumps were noted in blood smears from all these horses at each sampling point and were the likely cause of the low platelet count. The median platelet counts at 0 versus 60 h was still not significantly different after removal of the data points from these 3 horses. Red blood cell agglutination was noted in only 1 horse after treatment with UFH. Anemia, likely due to red blood cell agglutination, has been described as a consequence of UFH treatment (19, 25–28). Even though agglutination was not seen in most UFH-treated horses and the median hematocrit was unchanged from baseline values at 60 h, median results may obscure changes in individual horses. We thus examined changes in hematocrit in the individual horses before and after treatment. Two, 6 and 4 horses had a decrease in hematocrit with LMWH, UFH and placebo, respectively, and the median change in the hematocrit at 60 h was an increase of 0.02 L/L [2% in conventional units] (range, decrease of 0.02 to increase of 0.08 L/L) for LMWH, a decrease of 0.02 L/L (range, decrease of 0.07 to increase of 0.05 L/L) for UFH, and 0 L/L (range, decrease of 0.06 to an increase of 0.05 L/L) for placebo. These results do not support a substantial anemia associated with UFH therapy as used in this study. Few significant changes were seen in median biochemical test results before and after each treatment. Median serum urea nitrogen and creatinine concentrations decreased and increased, respectively, at 60 h (Table 3).

**Table 2:** Median (range) select automated hematologic results before treatment (T0) and 24 h after the last subcutaneous dose (T60) of placebo, unfractionated heparin (UFH) and low-molecular-weight heparin (LMWH) in 9 horses.

Test	Units	Placebo		UFH		LMWH		Reference interval
		T0	T60	T0	T60	T0	T60	
HCT	L/L	31 (38–47)	42 (33–46)	40 (37–47)	40 (31–44)	39 (37-46)	42 (36-48)	34–46
RBC	x 10^12^/L	7.8 (7.2–8.7)	7.7 (6.5–8.6)	7.4 (6.6–8.6)	7.4 (6.1–8.3)	7.3 (6.1–8.6)	7.1 (6.5–10.6)	6.6–9.7
WBC	x 10^9^/L	7.0 (5.7–10.2)	7.2 (5.2–9.6)	7.8 (6.0–9.4)	6.8 (4.4–10.5)	7.3 (5.4–9.9)	7.7 (7.1–8.9)	5.2–10.1
PMN	x 10^9^/L	4.4 (3.3–6.6)	4.1 (3.3–6.2)	4.7 (3.6–6.4)	3.8 (2.4–7.8)	4.4 (3.2–6.2)	4.5 (3.6–7.0)	2.7–6.6
LYMPH	x 10^9^/L	2.3 (1.6–3.5)	2.4 (1.5–3.2)	2.2 (1.2–3.2)	2.1 (1.4–3.3)	2.6 (1.5–2.9)	2.3 (1.6–3.6)	1.2–4.9
PLAT	x 10^9^/L	112 (30–209)	120 (32–176)	107 (51–176)	128 (95–180)	124 (94-197)	122 (22-194)	94–232

For all tests, there was no significant difference (p > 0.05) comparing T60 versus T0.

HCT, Hematocrit; RBC, Red blood cell count; WBC, White blood cell count; PMN, Absolute automated neutrophil count; LYMPH, Absolute automated lymphocyte count; PLAT, Platelet count.

**Table 3:** Median (range) select biochemical results obtained before treatment (T0) and 24 h after the last subcutaneous dose (T60) of placebo, unfractionated heparin (UFH) and low-molecular-weight heparin (LMWH) in 9 horses.

Test	Units	Placebo		UFH		LMWH		Reference interval
		T0	T60	T0	T60	T0	T60	
SUN	mmol/L	7.1 (5.7–8.6)	6.1* (5.0–8.6)	6.8 (5.7–9.3)	6.1* (4.6–8.6)	6.8 (5.4–8.9)	5.7* (4.6–7.1)	3.9–10.0
CREAT	μmol/L	115 (106–150)	123 (106–133)	106 (80–141)	115* (106–141)	106 (88–141)	115* (106–150)	53–141
SDH	U/L	5 (3–13)	3* (3–4)	4 (2–6)	3 (2–10)	3 (2–6)	3 (2–6)	0–11
GLDH	U/L	5 (3–14)	4 (3–8)	5 (3–6)	4 (3–6)	4 (3–6)	4 (3–8)	1–8
AST	U/L	279 (229–375)	277 (226–381)	295 (245–354)	309 (231–345)	274 (230–390)	295 (238–402)	199–374
GGT	U/L	14 (9–18)	14 (9–17)	16 (10–19)	17 (11–20)	15 (9–22)	14 (8–21)	8–29
TP	g/L	68 (64–75)	68 (64–73)	69 (65–72)	67 (65–77)	68 (62–76)	67 (63–74)	57–77
ALB	g/L	33 (30–34)	31 (29–36)	32 (30–35)	33 (29–37)	32 (30–35)	32 (29–35)	30–37
GLOB	g/L	37 (31–42)	35 (32–42)	37 (32–40)	36 (33–40)	35 (32–41)	35 (33–41)	24–44
SAA	mg/L	5 (5–6)	5 (5–5)	5 (5–7)	5 (5–1090)	5 (5–5)	5 (5–27)	0–20

* p < 0.05 comparing medians of T0 versus T60 within each treatment.

SUN, Serum urea nitrogen; CREAT, Creatinine; SDH, Sorbitol dehydrogenase; GLDH, Glutamate dehydrogenase; AST, Aspartate aminotransferase; GGT, Gamma glutamyl transferase; TP, Total protein; ALB, Albumin; GLOB, Globulins; SAA, Serum amyloid A.

**Figure 3:**
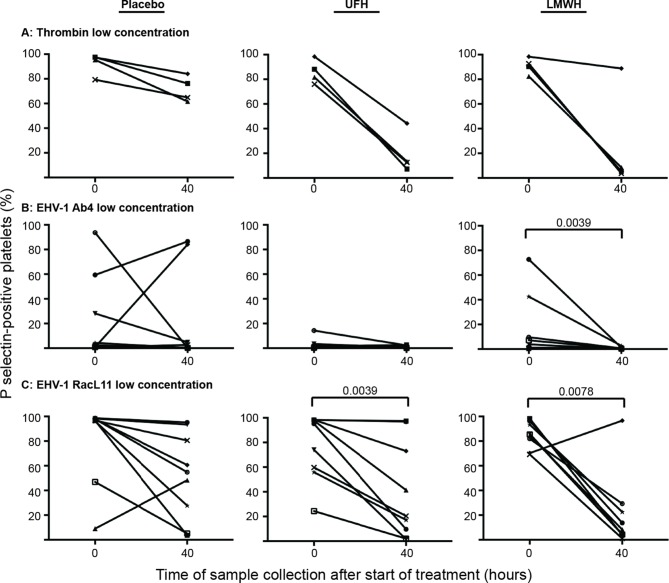
Changes in the percentage of platelets expressing P selectin in platelet-rich plasma of individual horses at 0 h (baseline) and 40 h after starting treatment (4 h after the last dose) with placebo (left column), unfractionated heparin (UFH, middle column) or low-molecular-weight heparin (LMWH, right column). Platelets were stimulated with low concentrations of thrombin **(A)**, 0.15 U/ml, *n* = 4) or the EHV-1 strains (1 × 10^6^ PFU/ml), Ab4 **(B)** and RacL11 **(C)**, for 10 min *ex vivo* (*n* = 9). Significant changes in the median percentage of platelets expressing P selectin at 40 h compared to 0 h are shown. Each horse has the same unique symbol at both time points for all treatments (horse 1: -□-; horse 2: -○-; horse 3: -⋆-; horse 4: -⧫-; horse 5: -X-; horse 6: -▾-; horse 7: -·-; horse 8: -▪-; horse 9: -▴-).

**Figure 4:**
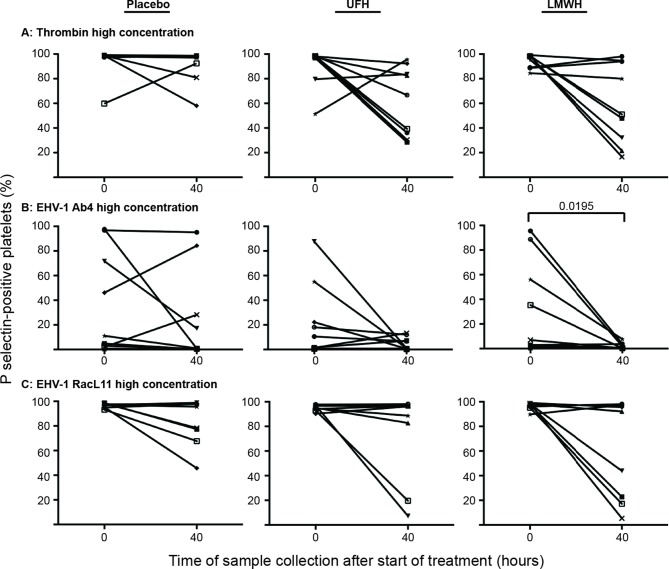
Changes in the percentage of platelets expressing P selectin in platelet-rich plasma of 9 individual horses at 0 h (baseline) and 40 h after starting treatment (4 h after the last dose) with placebo (left column), unfractionated heparin (UFH, middle column) or low-molecular-weight heparin (LMWH, right column). Platelets were stimulated with high concentrations of thrombin **(A)**, 0.5 U/ml) or the EHV-1 strains (5 × 10^6^ PFU/ml), Ab4 **(B)** and RacL11** (C)**, for 10 min *ex vivo*. Significant changes in the median percentage of platelets expressing P selectin at 40 h compared to 0 h are shown. Each horse has the same unique symbol at both time points for all treatments (horse 1: -□-; horse 2: -○-; horse 3: -⋆-; horse 4: -⧫-; horse 5: -X-; horse 6: -▾-; horse 7: -·-; horse 8: -▪-; horse 9: -▴-).

**Figure 5:**
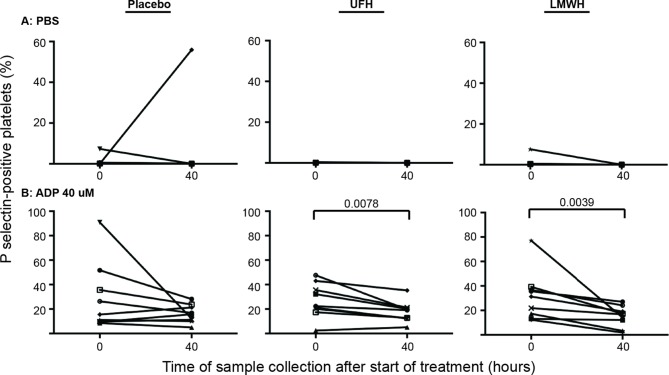
Changes in the percentage of platelets expressing P selectin in platelet-rich plasma of 9 individual horses at 0 h (baseline) and 40 h after starting treatment (4 h after the last dose) with placebo (left column), unfractionated heparin (UFH, middle column) or low-molecular-weight heparin (LMWH, right column). Platelets were exposed to the PBS negative control **(A)** or the ADP positive control **(B)**, 40 µM) for 10 min *ex vivo*. Significant changes in the median percentage of platelets expressing P selectin at 40 h compared to baseline (0 h) are shown. Each horse has the same unique symbol at both time points for all treatments (horse 1: -□-; horse 2: -○-; horse 3: -⋆-; horse 4: -⧫-; horse 5: -X-; horse 6: -▾-; horse 7: -·-; horse 8: -▪-; horse 9: -▴-). Note the Y-axis scale for PBS differs from that of ADP.

Individual horses showed substantial variability in baseline and post-treatment platelet activation in response to all stimuli (Figure 3 and Figure 4 for low and high thrombin and viral concentrations, respectively; Figure 5 for PBS and ADP controls; only 0 and 40 h shown). However, at the time of maximal measured anti-Xa activity (40 h), inhibition was most consistently observed among horses for platelets exposed to low concentrations of the various stimuli. In contrast, several horses demonstrated no evidence of drug-induced platelet inhibition with high concentrations of thrombin and EHV-1, with the higher concentrations of stimulants more readily inducing platelet activation (Figures 2–4).

**Figure 6:**
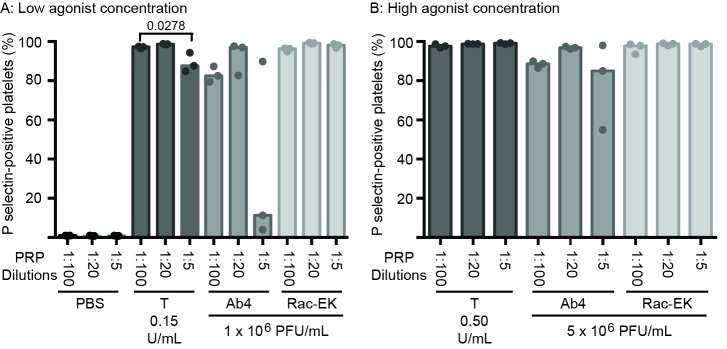
Changes in the median percentage of platelets expressing P selectin (with individual data points) in platelet-rich plasma (PRP) diluted 1:100, 1:20 and 1:5 in binding buffer. The diluted PRP was then exposed to low **(A)** or high **(B)** concentrations of thrombin (low concentration, 0.15 U/ml; high concentration 0.5 U/ml) and the EHV-1 strains (low concentration, 1 × 10^6^ PFU/ml; high concentration 5 × 10^6^ PFU/ml), Ab4 and RacL11, for 10 min *ex vivo*. Phosphate-buffered saline (PBS) served as a negative control for platelet stimulation. Blood was collected from the same horse on 3 different occasions for these experiments. Significant changes in the median percentage of P selectin-positive platelets between dilutions are shown.

In addition to within-horse variability in baseline platelet reactivity to EHV-1, we noted that the percentage of platelets expressing P selectin after exposure to low or high concentrations of the Ab4 strain were lower than what we have published previously (30). There are several possible explanations for this, including the use of different horses (with intrinsic variation in coagulation factor or inhibitor concentrations), the use of different lots of purified virus, or an effect of the change in protocol with decreased sample dilution. To address the latter possibility, we diluted PRP obtained from a single horse (not included in the study) 1:5, 1:20 and 1:100 in platelet buffer, before adding uniform concentrations of agonists. We found that the PRP dilution affected the platelet activation response, with fewer platelets expressing P selectin in the more concentrated samples (1:5 dilution), particularly after exposure to low concentrations of Ab4 as an agonist. In addition, there was more within-horse variability in platelet activation at the lower PRP dilutions of 1:5 and 1:20 with the Ab4 strain as an agonist. In contrast, platelet activation after exposure to RacL11 at both concentrations was largely unaffected by dilution of PRP in this particular horse (Figure 6). This data suggests that decreased inhibitor concentrations or lower total platelet numbers in the more dilute PRP samples yields more consistent platelet activation, particularly in response to the Ab4 strain, which was propagated in the rabbit kidney 13 cell line.

**Figure 7:**
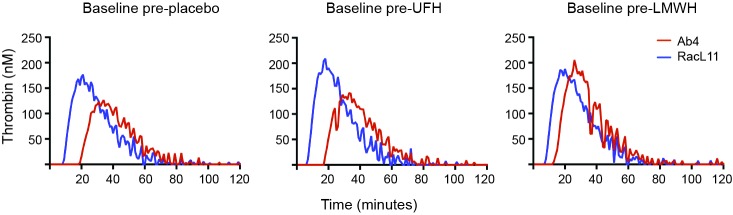
Representative curves showing the variation in baseline thrombin generation (nm thrombin generated over time in minutes) in platelet-poor plasma (PPP) from a single horse (horse 2) exposed *ex vivo* to high concentrations (5 × 10^6^ PFU/ml) of the EHV-1 viral strains, Ab4 and RacL11. Baseline samples were obtained before horse was given placebo, unfractionated heparin (UFH) or low-molecular-weight heparin (LMWH). The RacL11 strain had a shorter lag time and faster rate of thrombin generation than the Ab4 strain. In this particular horse, the RacL11 strain generated more thrombin than the Ab4 strain.

We have found that EHV-1 induces platelet activation via thrombin generation, which is mediated by tissue factor (17) presumably incorporated into the virus envelope as it buds from the propagating host cell membrane (18). We reasoned that the more consistent within- and between-horse platelet activation in response to exposure to the equine kidney cell-propagated virus, RacL11, was due to more effective triggering of thrombin generation with larger amounts of thrombin generated by equine host cell-derived tissue factor. To test this theory, we measured thrombin generation of baseline PPP samples from a subset of 5 horses in the study, using low and high concentrations of both strains of EHV-1 to initiate factor activation. The 5 selected horses showed substantial variability in their baseline platelet reactivity to one or more agonist (Table 4). Since the lower concentration of virus did not consistently generate thrombin, we calculated the median and range of thrombogram results from the PPP of the 5 horses for the high virus concentrations only, using the average of the 3 baseline values of each thrombogram parameter for each horse. We found that the equine kidney cell-propagated RacL11 strain yielded shorter median lag times (*p* = 0.063), a quicker median time to peak (*p* = 0.063), and faster rate of thrombin generation (*p* = 0.188) than the rabbit kidney cell propagated virus, Ab4 (Figure 7). However, the median peak thrombin concentration (*p* = 0.183) and overall thrombin generation (area under the curve, *p* = 0.625) were similar (Table 5). Considering that the virus was allowed to react with platelets for a maximum of 20 min in the platelet stimulation assay, the shorter lag and faster rate of thrombin generation with the equine kidney cell-propagated virus may partly explain the more reliable activation response due to attainment of a threshold amount of thrombin formation before quench dilution of the sample. The total amount of thrombin generated in each horses’ PPP did not entirely explain the extent of their platelet activation response to the viruses, as shown by the lack of a consistent association between the percentage of platelets expressing P selectin and AUC in the baseline samples of the individual horses (Tables 4–5).

**Table 4:** Percentage of platelets expressing P selectin in platelet-rich plasma (PRP) and total thrombin generation, expressed as area under the thrombin generation curve (AUC), in platelet-poor plasma (PPP) from a subset of 5 horses at all 3 baseline (0 h) time points (B1-3, before initiation of drug therapy*) in a heparin treatment trial.

Agonist	Concentration(PFU/mL)	P selectin-positive platelets (%)			AUC (nM thrombin × minute)		
		B1	B2	B3	B1	B2	B3
		Horse 2					
PBS		0.47	0.03	0.38	0	0	0
Ab4	1 × 10^6^	9.6	93.7	14.3	890	265	114
	5 × 10^6^	88.7	97.6	18.1	4,990	3,714	4,056
RacL11	1 × 10^6^	81.9	97.9	98.4	969	422	716
	5 × 10^6^	96.1	96.7	97.8	5,029	4,699	5,088
		Horse 3					
PBS		0.15	0.1	7.6	88	0	88
Ab4	1 × 10^6^	1.0	4.2	42.5	128	25	0
	5 × 10^6^	55.0	11.1	56.0	1,827	1,295	685
RacL11	1 × 10^6^	55.9	96.1	93.4	89	1	0
	5 × 10^6^	94.6	97.3	89.8	1145	1,124	634
		Horse 4					
PBS		0.2	0.0	0.1	0	0	0
Ab4	1 × 10^6^	3.1	0.9	0.9	54	2	54
	5 × 10^6^	22.1	46.1	3.0	2,919	2,465	3,170
RacL11	1 × 10^6^	98.4	97.8	70.3	72	2	203
	5 × 10^6^	90.3	95.2	99	3,143	2,906	3,685
		Horse 6					
PBS		7.3	0.1	0.3	0	0	7
Ab4	1 × 10^6^	28.1	1.2	3.4	7	26	505
	5 × 10^6^	71.7	1.2	87.6	1,913	2,130	2,611
RacL11	1 × 10^6^	98.3	85.9	74.0	0	95	552
	5 × 10^6^	97.1	98.2	97.1	1563	1,899	2,145
		Horse 7					
PBS		0.2	0.1	0.6	0	0	0
Ab4	1 × 10^6^	72.7	0.7	59.4	313	216	346
	5 × 10^6^	95.5	10.5	96.8	3,749	4,261	4,235
RacL11	1 × 10^6^	96.3	95.1	98.6	466	230	57
	5 × 10^6^	97.3	96.8	97.6	4,644	4,616	4,378

The same plasma samples (PRP and PPP) were exposed to PBS as a negative control and low (1 × 10^6^ PFU/ml) or high (5 × 10^6^ PFU/ml) concentrations of the EHV-1 strains, Ab4 and RacL11, propagated in rabbit and equine kidney cells, respectively, as a trigger for thrombin generation.

* Horse 2: B1 = Pre LMWH, B2 = Pre Placebo, B3 = Pre UFH.

Horse 3: B1 = Pre UFH, B2 = Pre Placebo, B3 = Pre LMWH.

Horse 4: B1 = Pre UFH, B2 = Pre Placebo, B3 = Pre LMWH.

Horse 6: B1 = Pre Placebo, B2 = Pre LMWH, B3 = Pre UFH.

Horse 7: B1 = Pre LMWH, B2 = Pre UFH, B3 = Pre placebo.

**Table 5:** Median (range) thrombin generation (thrombogram) results in platelet-poor plasma from time 0 (baseline) samples of a subset of 5 horses in a heparin treatment trial.

	EHV-1 strain	
Thrombogram result	Ab4	RacL11
Lag time (minutes)	21 (17–29)	13 (8–19)
Peak thrombin (nM)	108 (43–158)	102 (32–191)
Time to peak (minutes)	40 (29–46)	26 (18–40)
Velocity index (nM thrombin/minute)	3.5 (1.1–7.9)	6.3 (1.2–16.3)
Area under the curve(nM thrombin x minute)	2,851 (1,269–4,253)	3,025 (968c4864)

Thrombin generation was triggered by a high concentration (5 × 10^6^ PFU/ml) of the EHV-1 strains, Ab4 and RacL11, which were propagated in rabbit kidney 13 and equine kidney cells, respectively. The results from each horse were the average of 3 baseline samples. Median results for the 5 horses were not significantly different between Ab4 and RacL11 strains (p > 0.05).

## Discussion

Our study revealed that LMWH administered for 2 days to healthy horses at a dose of 80 U/kg q. 24 h resulted in an inhibition of *ex vivo* EHV-1 induced platelet activation. The inhibitory effect of LMWH was associated with the maximum measured anti-Xa activities ranging from 0.15 to 0.48 U/ml at 4 h after drug administration but did not persist through 24 h from the final dose. We did not measure anti-Xa activities or *ex vivo* platelet activation between 4 and 24 h after the last dose of LMWH to further characterize the change over time for these variables. A previous pharmacokinetic study of this LMWH dose showed that the mean anti-Xa activity remained above the therapeutic range for up to 10 h after single dose administration (23). Our study supports the use of more than once daily LMWH administration to sustain higher anti-Xa activities capable of suppressing platelet reactivity for a longer period of time (24). However, the dosing interval remains to be determined and increased frequency of drug dosing could result in excessive anticoagulant action and place the treated horse at risk of hemorrhage, as was noted in one LMWH-treated horse that developed a suspected jugular hematoma after venipuncture.

The low anti-Xa activities achieved in the UFH-treated horses, even at the expected time of peak activity [4–6 h after a single dose (21, 22)], would explain the lack of a consistent inhibitory effect of this form of heparin on EHV-1-induced platelet activation. At 4 h after the third and last maintenance dose of UFH, the median anti-Xa activities in this study were lower than the mean activity (around 0.13 anti-thrombin U/mL) obtained after administration of higher doses of UFH (loading dose of 150 U/kg and 3 twice daily doses of 120 U/kg) to 6 healthy horses (28). In addition, the mean anti-Xa activity of 25 horses with medical or surgical colic, that were given a loading dose of UFH of 150 U/kg followed by 125 U/kg twice daily, was higher after 2 days of treatment (around 0.35 anti-Xa U/mL) (19) than our maximum measured median activity. Our lower maximum anti-Xa activity may be related to the lower dose of UFH used in this study, however higher mean anti-Xa activities (0.253 U/ml) were reported after 2 days of treatment of 10 healthy horses given similar doses to that used herein (100 U/kg UFH twice daily) (26). The differences in the anti-Xa activity between this and previously published studies may be related to different reagents, calibrators or sensitivity of the activity assays, or individual horse variability in drug elimination, which can be high for UFH (19, 22, 26, 28). Nevertheless, it is possible that administering a higher loading and maintenance dose or the same dose of UFH over a longer period of time would achieve higher *in vivo* drug concentrations that could then inhibit EHV1-induced thrombin generation.

Similar numbers of horses treated with LMWH and UFH developed subcutaneous swellings. These localized cutaneous reactions are a known side effect with UFH but are reported to be less common or less severe with LMWH (19, 24–27). In our study, the observed swellings at injection sites for both heparins were mild and none were hot or painful. All resolved within 24 to 48 h and did not require specific treatment, as previously reported (19, 24). One horse apparently had excessive bleeding after venipuncture with placebo and LMWH injections. This was thought to be unique to this horse and unlikely treatment-related.

Red blood cell agglutination, anemia and thrombocytopenia has been observed in horses treated with UFH but not LMWH (19, 26–28). We noted red blood cell agglutination in 1 UFH-treated horse, but none of the LMWH- or placebo-treated horses. This agglutination did not appear to be of clinical consequence, however more severe changes may have been seen with higher doses or more prolonged treatment with UFH. The changes in median sorbitol dehydrogenase activity and median serum urea nitrogen and creatinine concentrations, although significant with some treatments, were mild and unlikely to be clinically relevant. Two horses had high serum amyloid A concentrations, one horse after UFH treatment and a second horse after the second placebo injection. Neither of these horses had obvious clinical signs of inflammation at the time of the increase in serum amyloid A concentrations and the high concentrations had resolved after the 10 day washout period, before administration of the next treatment, indicating transient inflammation. Data analyses with and without removal of the results of the horse with the fetlock wound did not alter any conclusions, so the results were included in this report.

In spite of the variable ability of heparin to reduce the treated horses’ platelet activation response to low concentration of virus, there was no change in the median percentage of P selectin-positive platelets after exposure to high concentrations of the RacL11 strain in the treated horses. These results suggest that neither of these heparin-based drugs will be completely effective at inhibiting EHV-1-triggered thrombosis in infected horses. Heparins were also less effective at inhibiting platelet activation response to high concentrations of thrombin. However more individual horses treated with LMWH and UFH showed some inhibition of platelet activation induced by a high concentration of thrombin compared to the high concentration of RacL11. The median percentage of activated platelets also decreased after stimulation with the high concentration of thrombin but not RacL11. This data suggests that the high concentration of RacL11 is generating more than 0.5 U/ml of thrombin. It is conceivable that high virus concentrations can be locally produced after efficient viral replication in endothelial cells and could result in localized thrombosis despite heparin therapy. In support of this possibility, neurologic disease was still observed in 1/31 horses treated with 25000 units of UFH twice daily for 3 days versus 7/30 untreated horses after an EHV-1 outbreak in a single facility in Germany (32). Alternatively, the neurologic disease in the UFH-treated horses may be due to neural injury that is unrelated to thrombosis, such as edema or the incited inflammatory response to virus replication (12). The results of the previously reported heparin treatment trial in a natural outbreak (32) and the study herein do support the use of heparin-based anticoagulants to help reduce the incidence or severity of neurologic disease in EHV-1-infected horses, however it is unlikely that this and other clinical syndromes related to EHV-1, including abortion, will be completely prevented by such treatments.

The RacL11 strain consistently induced stronger platelet activation than the Ab4 strain, regardless of time point and treatment. Thrombin generation assays performed on baseline samples in a subset of 5 horses revealed that this strain more quickly triggered initial thrombin formation and had a faster rate of thrombin generation. Since this strain was propagated in equine cell cultures, we attributed these findings to more effective binding of equine plasma Factor VII to the equine origin tissue factor that is likely incorporated into the virus envelope during propagation. The homologous equine tissue factor-Factor VII complex may more effectively interact with the other equine coagulation factors to initiate and amplify thrombin generation compared to the heterologous rabbit tissue factor-equine Factor VII complex. Alternatively, more tissue factor or procoagulant phospholipid may be expressed in the equine cell-propagated virus. In support of the latter theory, we did find that the equine kidney cells generated more activated Factor Xa, using an *in vitro* assay that assesses the activation of Factor X by exogenously added activated recombinant human factor VII in the presence of a tissue factor-bearing cell (16) (data not shown). Unfortunately, neither equine origin tissue factor nor Factor VII is available to test either of these theories more directly. We also did not have sufficient amounts of a single isolate of rabbit kidney cell-propagated RacL11 or equine kidney cell-propagated Ab4 to use in this study to directly compare different host-derived viruses. Regardless, we believe that the equine kidney-derived virus is a more appropriate mimic of *in vivo* replicating virus than the rabbit kidney-derived virus.

A significant albeit mild decrease in the median percentage of platelets expressing P selectin in response to ADP stimulation was observed 4 h after the last dose of LMWH and UFH. We attributed this to ADP-induced release of procoagulant intracellular platelet constituents, such as Factor V, which may result in a degree of thrombin generation in such samples.

There was substantial variability in baseline P selectin expression after stimulation with all agonists. In addition, an unexpected decrease in the median percentage of P selectin-positive platelets after stimulation with low concentrations of thrombin and RacL11 and both concentrations of Ab4 was seen at 40 h with the placebo. These results indicate the importance of having a placebo in such treatment trials. Nevertheless, comparing the change in individual results of the horses between 0 and 40 h showed more consistent and larger inhibition in horses given LMWH and, to a lesser extent, UFH than placebo. Only one LMWH-treated horse did not show any inhibition of platelet activation after exposure of PRP to the low concentration of RacL11 at 40 h. This horse had the lowest anti-Xa activity at this time point, suggesting that the lack of platelet inhibition was due to low levels of circulating anticoagulant drug. The high variability in the platelet activation assay was partly related to the dilution of PRP. We believe that the lower dilution of PRP used in this study is closer to the physiologic hemostatic status of the animal than the higher dilution of up to 1:150 used previously. Ideally, the test should be performed without dilution but this is not possible considering the need to overcome the citrate anticoagulant with exogenously added calcium.

The thrombin generation assay results also showed substantial differences among horses at baseline. This could not be attributed to an inadequate washout period, because some of the lowest results for thrombin generation in the individual horses occurred after placebo treatment. The variability observed in both platelet activation and thrombin generation assays at baseline and after treatment with placebo may be due to inherent biological variation in hemostasis in horses, as has been reported for viscoelastic-based hemostasis testing in horses (33) and for hemostasis testing in people (34). Because the total amount of thrombin generated did not explain the differences in the baseline platelet responses to agonists in individual horses, the biological variation is likely to be intrinsic to coagulation factors that trigger or drive thrombin generation, inhibitors of hemostasis, and platelets themselves (34). Thrombotic complications of EHV-1 cannot be predicted in individual horses and it is possible that horses that do suffer from EHV-1-induced abortion and neurological disease may be inherently more hypercoagulable or hyper-responsive to the virus on particular days and that these clinical syndromes may result from the combination of the infected horses’ innate physiologic responses and viral factors, such as the previously identified DNA polymerase polymorphism (35). In support of this theory, the virus glycoprotein D and host-derived cytokines have been recently associated with neuropathogenicity (36). It is also possible that the variable results in platelet activation and thrombin generation are due to pre-analytical variables, such as activation of hemostasis during sample collection. Even though we took great care during sample collection and handling, including “resting” samples for 30 min after harvesting PRP, samples from some horses did show platelet activation with no agonist (PBS negative control), indicating that such pre-analytical variables cannot be completely avoided.

In conclusion, the results of this heparin anticoagulant study show that LMWH is superior to UFH for inhibiting *ex vivo* EHV-1 induced platelet activation in PRP. More consistent anti-Xa activity levels and higher platelet inhibitory action was achieved with LMWH compared to UFH at the doses and for the duration given in this study. Our results support undertaking treatment trials of LMWH to reduce thrombosis-related sequelae in EHV-1-infected or exposed horses. However, because platelet-inhibitory activities of LMWH persisted for less than 24 h, the use of more than once daily injections should be evaluated. More frequent dosing, however, may increase adverse bleeding events and alter the risk benefit profile for certain patient populations, such as pregnant mares.

## Ethics Statement

The study was approved by the Institutional Laboratory Animal Care and Use Committee at Cornell University (protocol No. 2017-0030).

## Author Contributions

TS conceived and designed the study, collected blood samples, performed the red blood cell agglutination tests, did the flow cytometric and thrombin generation experiments, reviewed and interpreted the data, and wrote the manuscript; PS helped with study design and organization, prepared the virus and drugs, and performed some of the flow cytometric assays; MB helped with study design, data interpretation, and oversaw the anti-Xa activity assays; and TD and SN helped design the study and collected blood samples. All authors read and edited the manuscript.

## Conflict of Interest Statement

The authors declare that the research was conducted in the absence of any commercial or financial relationships that could be construed as a potential conflict of interest.

## Abbreviations

AUC: Area under the curve
LMWH: Low-molecular-weight heparin
PPP: Platelet-poor plasma
PRP: Platelet-rich plasma
UFH: Unfractionated heparin
